# Microvascular and Endothelial Dysfunction in Prediabetes

**DOI:** 10.3390/life13030644

**Published:** 2023-02-25

**Authors:** Stamatina Lamprou, Nikolaos Koletsos, Gesthimani Mintziori, Panagiota Anyfanti, Christina Trakatelli, Vasileios Kotsis, Eugenia Gkaliagkousi, Areti Triantafyllou

**Affiliations:** 1 Third Department of Internal Medicine, Papageorgiou General Hospital, Aristotle University of Thessaloniki, 56429 Thessaloniki, Greece; 2 Unit of Reproductive Endocrinology, 1st Department of Obstetrics and Gynecology, Papageorgiou General Hospital, Aristotle University of Thessaloniki, 56429 Thessaloniki, Greece; 3 Second Medical Department, Hippokration Hospital, Aristotle University of Thessaloniki, 54642 Thessaloniki, Greece

**Keywords:** prediabetes, endothelial dysfunction, cardiovascular disease, skin microcirculation, retinopathy, microalbuminuria

## Abstract

Prediabetes is a significant metabolic status since there is high potential for future progression of diabetes mellitus (DM). People with prediabetes are at increased risk of cardiovascular disease (CVD) and mortality. Endothelial and microvascular dysfunction is considered a key step towards the development and progression of CVD. Importantly, endothelial and microvascular dysfunction can be detected and monitored using non-invasive procedures in peripheral organs and tissues, including the retina, kidney, skin and skeletal muscle. Structural and functional alterations of the microvasculature have been consistently documented in the above microvascular beds in patients with diabetes mellitus. In contrast, such alterations remain understudied in prediabetes, but are currently receiving attention as markers of subclinical and future CVD. The aim of this review is to summarize available evidence regarding the presence of subclinical microvascular and endothelial dysfunction in prediabetes and their impact on cardiovascular risk.

## 1. Introduction

Prediabetes is recognized as a significant metabolic status, and as a precursor of diabetes mellitus (DM) and its complications. It is estimated that approximately 25% of people with prediabetes will develop DM in the next 3–5 years and 70% at some point in their life [1]. The prevalence of prediabetes has increased globally, and it is expected that more than 470 million people will develop prediabetes by 2030 [2]. According to the latest epidemiological report of the CDC (Center for Disease and Prevention) in 2020, 34.5% of the adult population of the United States (88 million Americans) had prediabetes, while 10.5% of the population were diagnosed with DM [3].

The prevalence of prediabetes depends on the definition used. According to the American Diabetes Association (ADA), the definition of prediabetes is based on having any one of the three following criteria: Impaired Fasting Glucose (IFG) defined as fasting plasma glucose between 100–125 mg/dl, Impaired Glucose Tolerance (IGT) defined as two-hour glucose between 140–199 mg/dl during a 75-g oral glucose tolerance test (OGTT), or a plasma glycosylated haemoglobin (HbA1c) between 5.7–6.4%. However, in the presence of anaemia, chronic kidney disease, haematological diseases and other systemic diseases, HbA1c measurement is unreliable and its use as a diagnostic tool is limited [4,5,6].

Patients with established DM are at high risk for cardiovascular disease (CVD) [7]. Functional and structural microvascular damage occurs in patients with DM and often precedes the development of complications, such as advanced diabetic retinopathy, kidney failure and overt CVD [8]. Early, subclinical changes of micro- and macrocirculation may also be present in prediabetes. Identification of such alterations may be of crucial importance, as they could facilitate the early detection of vascular damage and contribute to the prevention of the above-mentioned DM-related complications [9]. Therefore, this narrative review aims to summarize the existing literature providing evidence on the presence of subclinical microvascular alterations in patients with prediabetes. In addition, their usefulness in terms of cardiovascular risk assessment will be critically evaluated. For this purpose, a PubMed search was performed to identify relevant articles published in English, using the following medical terms: “prediabetes”, “endothelial dysfunction”, “microvascular alterations”, “cardiovascular disease”, “skin microcirculation”, “retinopathy”, and “microalbuminuria”.

## 2. Prediabetes and Cardiovascular Disease

People with prediabetes have an increased risk for ischemic stroke or acute myocardial infarction (MI), as well as a 2.2-fold increased all-cause mortality compared to healthy control groups [9,10,11]. Both IFG and IGT have a predictive role for CVD, independently of the presence of diabetes, even though results are more consistent for IGT. The HOPE study showed that the risk of cardiovascular disease (MI, stroke, cardiovascular death) increased by almost 9% for every 1 mmol/L of FPG rising, and this relationship remained significant independently of age, sex, blood pressure, hyperlipidaemia, waist–hip ratio and intake of ramipril, in both diabetic and non-diabetic participants [12]. In the DECODE study, a collaborative prospective study of 22 cohorts in Europe with baseline glucose measurements for 29,714 subjects, aged 30–89 years, patients with IFG did not have an increased risk of death compared with those with normal FPG, even though IGT was better associated with CVD mortality [13]. Similar results were demonstrated by the Framingham Offspring and Funagata Diabetes studies, in which IGT, but not IFG, was identified as an independent risk factor for CVD and mortality, respectively [14,15]. In the Hoorn study, during an 8-year follow-up and after exclusion of patients with newly diagnosed DM or pre-existing CVD, IGT was also associated with an increased risk of all-cause [Relative Risk, RR: 2.2 (1.11–4.33)] and CVD mortality [RR: 3.0 (1.08–8.30)], even after adjustment for known CVD risk factors [16].

Qiao et al. have also shown that IGT is a strong prognostic indicator for the occurrence of stroke and cardiovascular events [17]. A prospective study conducted on patients without DM suffering from acute MI showed that 35% of them had IGT at discharge, while after three months, 31% of these patients had already developed DM [18]. Another prospective multicentred European study, involving approximately 2000 patients with acute coronary heart disease or stable angina, showed that 36% had IGT and 22% had undiagnosed DM [19]. A meta-analysis of 53 cohort studies with a large sample size (comprising 1.611.339 individuals) showed that prediabetes defined as IFG (according to ADA) or IGT was associated with a higher risk of CVD (RR 1.13, and 1.3 for IFG and IGT, respectively), coronary heart disease (1.1 and 1.2, respectively), stroke (1.06 and 1.2, respectively) and all-cause mortality (1.13 and 1.32, respectively), in comparison to normoglycemia. Raised HbA1c was also associated with an increased risk of CVD and coronary heart disease (1.21 and 1.15, respectively), but no statistically significant association was found between stroke and all-cause mortality [11]. Finally, the Rotterdam study, using 1007 patients with acute stroke (83.3% ischemic), showed that patients with IFG had a poorer prognosis for complete restoration of their mobility and were less likely to return home immediately after being discharged from the hospital compared to normoglycemic patients [20].

Regarding peripheral arterial disease (PAD), there is a well-established relationship with DM, while there are limited studies in individuals with prediabetes [21]. Moreover, DM is one of the most common causes for lower limb amputation worldwide [22]. However, existing data do not support an increased prevalence of PAD among individuals with prediabetes [23,24,25,26]. To this end, in the study of Silbernagel et al., the prevalence of prediabetes did not differ between individuals with PAD (assessed by symptoms consistent with PAD in combination with ultrasound findings) and the control group [24]. Additionally, Faghihimani et al. have shown that the ankle–brachial index was similar between individuals with prediabetes compared to control individuals [23].

Therefore, it has been established that the relationship between CVD and IGT is well documented, unlike the relationship between CVD and IFG. As glucose levels are not concluded in the cardiovascular risk scores, it is very important for health professionals to be alerted in the assessment, diagnosis and treatment of individuals with glucose levels higher than normal without exceeding thresholds for diabetes.

## 3. Endothelial Dysfunction in Prediabetes

The endothelium is considered to be an active metabolic organ that regulates vascular tone through the production of vasoactive substances such as nitric oxide (NO). Endothelial dysfunction is defined as the loss of vasodilator, anticoagulant and anti-inflammatory properties of endothelium and the predominance of mechanisms that promote vasoconstriction, thrombosis and inflammation in the arterial wall due to decreased NO availability [27,28]. Hence, the alterations observed in vascular walls lead to atherosclerosis predisposing to CVD. Therefore, endothelial dysfunction is considered a precursor of CVD and has been documented in patients with hypertension and high CVD risk [29]. Furthermore, endothelial dysfunction is increasingly being recognized as the primary pathophysiological process in novel clinical entities such as COVID-19 [30].

Insulin resistance and hyperglycaemia are predominantly involved in atherosclerotic vascular changes and the pathogenesis of macrovascular complications of prediabetes. Insulin resistance is related to the reduced function of glucose transporter GLUT-4 and the activity of NO, which is the main regulator of endothelial function, as well as the triggering thrombogenic processes. NO plays multiple roles in vascular physiology, such as vasodilation, regulation of vascular smooth muscle proliferation and expression of cellular molecules that are involved in the formation of atherosclerotic plaque. NO also locally inhibits platelet aggregation. In states of insulin resistance, reduced availability of NO leads to impaired endothelial function. At the same time, hyperglycaemia increases the production of inflammatory and vasoconstrictors resulting in endothelial vascular damage [31,32]. These mechanisms are considered to comprise an early stage in the pathophysiology of CVD [27,28].

Indicators of inflammation, oxidative stress and haemostasis used as biochemical markers of endothelial dysfunction in patients with DM include the intracellular adhesion molecule-1 (ICAM-1), the adhesion molecule of vascular cells-1 (VCAM-1), P-selectin, E-selectin, Asymmetric Dimethylarginine (ADMA), oxidized LDL particles and endothelin-1 [33,34]. On the other hand, vascular markers of endothelial dysfunction are based on the stimulation of NO production after mechanical (ischemic ligation) or vasoactive stimuli (administration of acetylcholine), and the measurement in changes in blood flow or vessel diameter. The most widely used, non-invasive method for assessing endothelial function in larger conduit arteries is flow-mediated dilation (FMD) of the brachial artery. Peripheral arterial tonometry (EndoPAT) measures endothelial function in the microvasculature using finger plethysmography to quantify pulsatile arterial volume changes [27,35,36].

There are few data regarding the presence of endothelial dysfunction in patients with prediabetes, using mainly biochemical indices. In a cohort study that tested the likelihood of developing DM in prediabetic and normoglycemic individuals based on serum VCAM-1 and ICAM-1, individuals with prediabetes were found to have higher concentrations of these indicators than control individuals [37]. Similar results were observed in the study of Wang et al. In fact, VCAM-1 and ICAM-1 were positively correlated with indicators of aortic stiffness [38]. In another small study in women of reproductive age with prediabetes, serum ADMA levels were used as an indicator of endothelial dysfunction and were found to be significantly associated with both elevated HbA1c and decreased FMD [39]. A few studies showed the presence of endothelial dysfunction and oxidative stress using specific serum and urine biomarkers (malondialdehyde, superoxide dismutase, urinary 8-hydroxy-2-deoxy-guanosine) [40,41]. Similarly, there are only a few studies investigating microvascular endothelial dysfunction by vascular markers in individuals with prediabetes. Gupta et al. studied endothelial dysfunction using EndoPAT in healthy obese people with elevated FPG levels and showed that these individuals tended to develop prediabetes and had a reduced reactive hyperaemia index (RHI) compared to control individuals [42].

To summarize, there is limited data concerning endothelial dysfunction in prediabetes, mainly using biochemical markers and some using vascular methods (either EndoPAT-microvascular or FMD-macrovascular), and all showing that endothelial dysfunction is present even in prediabetes. However, larger, prospective studies are needed to confirm the above-mentioned findings, explore possible underlying mechanisms and investigate the role of endothelial dysfunction in macro- and microvascular alterations in prediabetic patients. Moreover, assessment of endothelial function in individuals with prediabetes, using well-established vascular (FMD, EndoPAT) and biochemical (ADMA, VCAM-1, ICAM-1) markers could add context in clinical practice.

## 4. Prediabetes and Microcirculation

The term microcirculation refers to the circulation in vessels with diameter <150 μm, including the small arteries and veins, as well as the capillaries. The main function of microcirculation is to ensure the provision of nutrients and oxygen to tissues. It also regulates hydrostatic pressure at the level of capillaries and blood flow, and consequently, it helps in the regulation of blood pressure through the increase of peripheral resistance [43].

Several non-interventional vascular methods (Table 1) have been developed for the evaluation of peripheral microcirculation in divergent vascular beds, including the retina, kidney, skin and muscle tissue.

### 4.1. Prediabetes and the Retina

The retina offers an easy window to study the human microcirculation. Diabetic retinopathy (DR) is the most common microvascular complication of DM and remains the main cause of blindness inworking-age population. One third of patients with DM show signs of retinopathy and one third of them develops severe damage, which could potentially lead to blindness [44]. In the meta-analysis by Yau et al., concerning the global prevalence of DR, it was demonstrated that 35% of patients with DM will develop some type of retinopathy (proliferative in 7% of the cases) [45]. Similarly, the Wisconsin study highlighted that the 10-year incidence of DR was 74% for all patients with DM who participated [46].

In addition to the devastating effects on patients’ vision, the presence of DR is an independent risk factor for the development of CVD. Even mild retinopathy has been associated with a high risk for stroke, coronary heart disease and heart failure, as diabetic retinopathy is indicative of the presence of TOD in patients with DM [47,48]. However, in the Gutenberg study involving 5000 participants with prediabetes, no association between retinopathy and cardiovascular risk factors was detected [49].

Pathogenetic mechanisms that are implicated in DR have not yet been clarified. They are categorised as biochemical and vascular. The main biochemical factor is the vascular endothelial growth factor (VEGF). In diabetic retinopathy, retinal hypoxia induces over-expression of VEGF, which acts as a mitogenic agent of endothelial capillary cells. As a result, the permeability of the blood-retinal barrier increases and, finally, causes macular oedema. VEGF is also responsible for neovascularization, as it induces the proliferation of capillary endothelial cells of retina [50].

**Figure 1:**
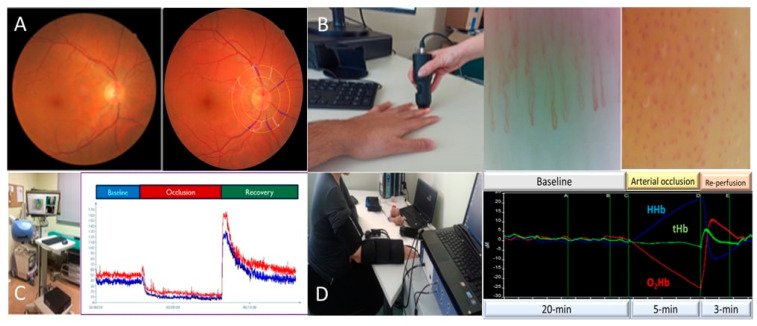
Indices of microvascular assessment. (**A**) Non-mydriatic digital fundus photography and imaging of retinal vessels. (**B**) Nailfold video capillaroscopy (NVC), examination and a representative image obtained during NVC. (**C**) Recording of skin microvascular reactivity during arterial occlusion and re-perfusion using Laser Speckle Contrast Analysis. (**D**) Near infrared spectroscopy—representative data of skeletal muscle oxygenation during arterial occlusion and after re-perfusion. HHb: Deoxygenated haemoglobin, tHb: Total haemoglobin, O_2_Hb: Oxygenated haemoglobin.

Abnormalities of the mean diameters of the retinal arterioles and venules, i.e., narrowing and widening, have emerged as novel vascular biomarkers indicative of individual cardiovascular risk and future onset of cardiovascular diseases, including hypertension [51]. A decade ago, our group showed that subtle alterations of the retinal microvascular diameters (Figure 1A) may even identify divergent hypertension phenotypes [52] with subsequent studies documenting the presence of altered retinal microvascular diameters in other high risk populations [53]. In contrast, there is still limited evidence regarding retinal vessel alterations in patients with prediabetes. There are few studies confirming the existence of retinopathy in prediabetic patients [54], with venular dilation being the main finding [55,56]. A recent cohort study showed that decreased macular thickness and retinal arterial stenosis were the main findings in the prediabetic group compared to the normoglycemic group [57].

However, studies investigating the relationship between the diameter of the retinal vessels and the risk of developing prediabetes and DM in the future have shown conflicting results. More specifically, in the Rotterdam study, a positive correlation in univariate analysis identified between decreased arteriovenous ratio (AVR) and the risk of IFG [OR:1.29 (1.13–1.46)], which however disappeared after adjusting for confounding factors [OR:1.14 (0.98–1.32)]. In this study, the risk of IFG and DM with AVR was thought to be due to the venular dilatation rather than the arteriolar narrowing [58]. Similarly, in the “Blue Mountains” study, a correlation between the retinal venular dilation and the development of IFG was found, mainly in middle-aged people, while the diameter of arteries did not show a significant correlation [59]. In contrast, the AusDiab study showed that only the retinal arteriolar narrowing was associated with a higher risk of developing DM [60]. Similar results were observed in both the ARIC sub-study and the Wisconsin study [61,62]. In the latter, the risk of developing DM was three times higher in individuals with retinal artery stenosis and co-existed hypertension, compared to the normotensive participants without arteriolar narrowing [OR:3.41 (1.66–6.98)] [61].

Some very recent studies documented retinal vascular changes using optical coherence tomography angiography (OCTA) in prediabetic individuals compared to control individuals. The OCTA is a non-invasive fundus angiography imaging technique for assessing retinal vascular disease. Xu et al. showed that some parameters of the OCTA, more specifically, the size of focal avascular zone and the macular vessel diameter, were larger in the prediabetic patients compared to the control group. Moreover, the vessel area density in superficial macular area decreased in prediabetes [63]. In accordance with this study, Ratra et al. found that the decreased vessel diameter was positively correlated with HbA1c [64]. Another study from Ratra’s group, focusing on prediabetic individuals, found no difference in focal avascular zone parameters, even though the central foveal thickness significantly decreased in prediabetes compared to the control [65]. Similarly, Arias et al. found no alteration in focal avascular zone area, but pointed out that perfusion density and vascular length density decreased in prediabetic people compared to the control [66]. On the other hand, Peng et al. identified some neuroretinal changes regarding the thickness of macula and the peripapillary retinal nerve fiber layer using both OCT and fundus fluorescein angiography, even though microvascular alterations were not detected in prediabetic individuals [67].

Another novel method for assessing retinal vascular dysfunction is the flickering light stimulus. In healthy individuals, the flickering light stimulus normally causes an increase of the retinal blood flow and blood vessel diameter, whereas in diseases, such as DM retinal vasodilation, they decrease. Lott et al. examined retinal vascular dilation responses to flicker in a study including a prediabetic, a diabetic and a control group, finding similar attenuated vasodilator responses in prediabetic and diabetic patients compared to the control group. These findings remained unchanged even after the adjustment for age, blood pressure and body mass index (BMI) [68].

### 4.2. Prediabetes and Albuminuria

Measurement of urine albumin excretion is a simple assessment tool of renal microvascular function. Albuminuria is defined as the urine albumin-to-creatinine ratio (ACR) ≥ 30mg/g [69]. Although the older terms, microalbuminuria (MAU) defined by ACR 30–299mg/g and macroalbuminuria (ACR ≥ 300mg/g), should be avoided according to the ADA guidelines, many studies still apply these terms [70]. In the same way, the normal ranges of 2.5−25 mg/mmol for males and 3.5−35 mg/mmol for females are still used [71]. MAU is a well-documented risk factor for cardiovascular morbidity and mortality. Patients with MAU are at high risk for acute coronary heart disease, stroke and peripheral arterial disease [71,72].

There are few data confirming the occurrence of MAU in prediabetes. The AusDiab study, including more than 10.000 participants, showed that the prevalence of MAU in IGT is 9.9%, in IFG 8.3%, and more than double in patients with diabetes (both type 1 and 2) [73]. It is worth mentioning that 30% of individuals with newly diagnosed DM already had some degree of kidney damage. This fact suggests that the effects of hyperglycaemia on the kidney may occur in the early stages, even before glucose levels reach diabetic ranges. A meta-analysis of nine cohort studies, including over 180,000 participants, showed that prediabetes was linked to an increased risk of renal dysfunction after adjustment for established risk factors [RR:1.11 95%CI (1.02–1.21)] [74].

There is further evidence that the presence of MAU in prediabetes could be an early indicator for the development of DM. A 10-year cohort study conducted in participants with MAU without DM showed that people with MAU were more likely to develop DM, even after adjustment for the presence of prediabetes [75]. Similarly, a Chinese cohort study showed that MAU was associated with increased risk of diabetes after a three-year follow-up in populations with normal glucose tolerance and impaired glucose regulation. Nevertheless, MAU did not remain a significant predictor of DM after adjustment for hypertension [76]. Moreover, Bahar et al. showed that people with prediabetes and albuminuria had a four-fold higher risk of developing DM compared to those with prediabetes without albuminuria [77]. In contrast, a study conducted in the United States did not find an independent predictive role of microalbuminuria for developing DM in obese patients with pre-diabetes [Hazard ratio, HR:0.98 (0.91–1.06)] [78].

It remains unclear whether other risk factors such as hypertension are also involved in the presence of MAU in prediabetes. A prospective, population-based, cohort study conducted in Netherlands with over than 6000 obese (BMI ≥ 27) participants showed that the prediabetes and newly diagnosed DM were associated with increased MAU [OR:1.6 (0.9–2.7) and OR:2.8 (1.5–5.4) respectively]. Moreover, FPG [OR:1.21 (1.04–1.42)] and HbA1c [OR:1.36 (1.00–1.86)] were positively associated with MAU. On the other hand, after adjustment for confounding risk factors (age, BMI, hypertension, smoking), the association between MAU and prediabetes did not remain statistically significant [79]. Similar studies found a positive correlation between microalbuminuria and the occurrence of prediabetes [80,81], whereas Kim et al. showed that this correlation is rather influenced by coexistence of hypertension in these individuals [OR:0.77 (0.55–1.09)] [82].

In conclusion, most data advocate that there is a positive association between prediabetes and some degree of renal dysfunction, in terms of kidney microvascular dysfunction. Thus, it might be possible that screening for microalbuminuria in individuals with prediabetes may lead to early detection and interventions resulting in fewer new cases of renal dysfunction.

### 4.3. Prediabetes and Skin—Muscle Microcirculation

The most widespread method of estimating skin microcirculation is nailfold capillaroscopy (Figure 1B). It is a non-invasive method that allows the imaging of capillaries by a stereomicroscope, usually applied to the fingernail bed. Capillaroscopy has been mainly used in rheumatic diseases, such as systemic sclerosis, systemic lupus erythematosus and rheumatoid arthritis [83,84,85]. In recent years, scientific interest has turned to the study of dermal microcirculation disorders which are involved in the pathophysiology of CVD. Although functional impairment can be detected by means of nailfold video capillaroscopy, assessment of morphological abnormalities is more frequently applied both in research and in clinical practice. Structural alterations of skin microcirculation have been consistently observed in patients with hypertension, with a decrease in the number of capillaries per field of vision being the main finding [86,87]. This finding has been demonstrated in other high cardiovascular risk populations, such as those with rheumatoid arthritis [88].

Studies on skin microcirculation showed that qualitative morphological changes in capillaries (tortuosity, absence of vessels, capillary dilation, irregular shape) were more commonly present in patients with type 2 DM than in individuals without type 2 DM history [89,90]. Furthermore, studies in patients with type 2 DM have shown that capillary abnormalities positively correlated with the occurrence of other microvascular complications of the disease [91,92,93]. Similarly, in the study of Kuryliszyn-Moskal et al., qualitative changes in capillaries were more common in patients with type 1 DM than in healthy control patients and positively correlated with indicators of endothelial dysfunction [94].

To date, there is no study investigating dermal capillaroscopy in patients with prediabetes. However, there are data from small studies in healthy populations, identifying capillary changes of the skin in potentially precursor forms of DM, such as individuals with insulin resistance and increased glucose. More specifically, a study by Irving et al. in young healthy men studying changes in skin microcirculation based on blood pressure levels and insulin resistance, showed that individuals with higher FPG concentrations had decreased capillary density and increased flow rate [95]. Another study in a healthy population showed that individuals with greater insulin sensitivity had higher capillary density in the examined limb after removal of the occlusive cause [96].

Laser Speckle Analysis (LASCA) is a non-invasive method for assessing skin microvascular function (Figure 1C). LASCA represents an evolution of the older laser doppler flowmetry techniques, but providing better spatial resolution. It enables assessment of microvascular perfusion in a larger tissue area and with higher reproducibility. The method is based on the speckle phenomenon to create dynamic two-dimensional maps of skin microvascular perfusion and visualize blood flow in real time with high spatial and temporal resolution. Microvascular responsiveness can be assessed using various stimuli, such as iontophoresis with acetylcholine or post-ischemic forearm skin reactive hyperaemia, which is the most commonly used. The first studies included mainly patients with rheumatic diseases [97,98,99,100,101]; nevertheless, in recent years, it has been applied in patients with CVDs [102,103].

Regarding patients with DM, Matheus et al. showed that microvascular reactivity of patients with type 1 DM was significantly affected compared to healthy individuals based on the response of the skin vessels’ microcirculation in various stimuli (iontophoresis with acetylcholine, ischemia) [104]. It has also been used for the estimation of the extent of skin lesions in foot ulcers of people with type 2 DM [105].

More evidence is available regarding the use of near infrared spectroscopy (NIRS) in patients with DM. The NIRS method (Figure 1D) estimates tissue oxygenation and provides information about indicators of tissues’ local oxygen consumption, as well as their blood flow. This technique is non-invasive, easy to use and with good reproducibility, emerging as a valuable tool for assessing microvascular function and dysfunction. Recently, Dipla et al. showed that women with gestational DM, exhibited a blunted muscle oxygenation and microvascular reactivity compared with women with uncomplicated pregnancies. These changes also showed a positive correlation with aortic stiffness (as estimated by pulse wave velocity) and 24-h blood pressure measurements [106]. Furthermore, a recent study of Townsend et al. using NIRS, showed that insulin resistance, which is considered to be a predisposing factor of DM, is associated to reduced microcirculatory response to induced ischemia based on tissue oxygenation parameters [107]. Soares et al. studied vascular responsiveness after an oral glucose challenge using NIRS combined with a vascular occlusion test in a small group of healthy individuals. They found that there were differential responses regarding the oxygen saturation, which corresponds to vascular adjustment to hyperglycaemia [108]. As far as we know, there is no data in the literature regarding the use of the NIRS method in prediabetes.

## 5. Conclusions

In conclusion, individuals with prediabetes are at increased risk of CVD in analogy with DM. Subclinical vascular changes, easily assessed by non-invasive methods, occur in prediabetes and probably anticipate the development of DM. Endothelial dysfunction is considered as the major underlying pathophysiological process related to vascular injury in prediabetes, and eventually resulting in TOD and clinically evident CVD manifestations. Nevertheless, the identification of subclinical microvascular alterations in divergent vascular beds represents a research hotspot in prediabetes, as relevant studies have often almost exclusively focused on the description of such lesions in DM.

While the debate as to whether prediabetes deserves targeted identification and clinical intervention is still ongoing, it can be hypothesized that lifestyle and/or pharmaceutical interventions targeting the reversal of early microvascular alterations might be accompanied by a reduction in cardiovascular risk associated with prediabetes. Appropriately designed studies are eagerly warranted to shed light on the clinical significance of microvascular endothelial dysfunction in prediabetes. In the meantime, healthcare professionals should be alert to effectively identify and monitor people with impaired glucose levels.

## Figures and Tables

**Table 1:** Non-interventional vascular methods for the evaluation of peripheral microcirculation in divergent vascular beds.

Peripheral Organ/Tissue	Non-Invasive Methods for Microvascular Assessment
Retinal microvasculature	Retinal photography, for the qualitative and quantitative evaluation of the retinal microvasculature (e.g., evaluation of retinal microvascular diameters)
	Optical coherence tomography angiography, for assessing with high accuracy the retinal vessels and the macula area
	Flickering light stimulus, to assess microvascular responses (specifically, in retinal blood flow and diameters)
Renal microvascular injury	Urinary albumin excretion (UAE), as an index of renal glomerular dysfunction
Skin microvascular network	Nailfold capillaroscopy and video capillaroscopy, for the qualitative and quantitative assessment of the dermal microcirculation (e.g., dermal capillary rarefaction)
	Laser doppler flowmetry (LDF), for the evaluation of dermal microvascular reactivity
	Laser Speckle Contrast Imaging (LSCI), as an evolution of older LDF techniques
	Peripheral arterial tonometry (PAT), for the evaluation of peripheral arterial vascular tone on the finger
Skeletal muscle microvasculature	Near infrared spectroscopy (NIRS), for monitoring of regional tissue oxygenation

## Data Availability

Data availability is not applicable because this study is based exclusively on published literature.
